# Preparation and Properties of Electrospun Poly (Vinyl Pyrrolidone)/Cellulose Nanocrystal/Silver Nanoparticle Composite Fibers

**DOI:** 10.3390/ma9070523

**Published:** 2016-06-28

**Authors:** Siwei Huang, Ling Zhou, Mei-Chun Li, Qinglin Wu, Yoichi Kojima, Dingguo Zhou

**Affiliations:** 1 College of Materials Science and Engineering, Nanjing Forestry University, Nanjing 210037, China; huangsi.wei@163.com; 2 School of Materials Science and Engineering, South China University of Technology, Guangzhou 510640, China; zhoulingscut@gmail.com; 3 School of Renewable Natural Resources, Louisiana State University Agricultural Center, Baton Rouge, LA 70803, USA; MLi@agcenter.lsu.edu; 4 Department of Environment & Forest Resources Science Faculty of Agriculture, Shizuoka University, Shizuoka 422-8529, Japan; aykojim@ipc.shizuoka.ac.jp

**Keywords:** electrospun, poly (vinyl pyrrolidone) (PVP), cellulose nanocrystals, silver nanoparticles, rheology, antimicrobial properties

## Abstract

Poly (vinyl pyrrolidone) (PVP)/cellulose nanocrystal (CNC)/silver nanoparticle composite fibers were prepared via electrospinning using *N*,*N*′-dimethylformamide (DMF) as a solvent. Rheology, morphology, thermal properties, mechanical properties, and antimicrobial activity of nanocomposites were characterized as a function of material composition. The PVP/CNC/Ag electrospun suspensions exhibited higher conductivity and better rheological properties compared with those of the pure PVP solution. The average diameter of the PVP electrospun fibers decreased with the increase in the amount of CNCs and Ag nanoparticles. Thermal stability of electrospun composite fibers was decreased with the addition of CNCs. The CNCs help increase the composite tensile strength, while the elongation at break decreased. The composite fibers included Ag nanoparticles showed improved antimicrobial activity against both the Gram-negative bacterium *Escherichia coli* (*E. coli*) and the Gram-positive bacterium *Staphylococcus aureus* (*S. aureus*). The enhanced strength and antimicrobial performances of PVP/CNC/Ag electrospun composite fibers make the mat material an attractive candidate for application in the biomedical field.

## 1. Introduction

Electrospun technology is widely used to form high-quality and well-defined fibers with submicron or nanoscale diameters. The resultant fibers have unique properties, i.e., high surface area-to-volume ratio, small pore sizes, high porosity, and the potential for controlled release of active materials [1,2]. Moreover, the electrospinning process provides a significant compromise, considering throughput and the control of size and shape that could be tuned by proper control of electrostatic forces [3]. The critical factors affecting the electrospinning process and the nanofiber morphology can be divided into four kinds: structural properties of polymer (molecular weight and tacticity), polymer solution parameters (concentration, electrical conductivity, viscosity, and surface tension), processing conditions (voltage, spinning distance, feed rate, and nozzle geometry), and ambient parameters (temperature, atmosphere pressure, and relative humidity) [4]. The average diameter of the electrospun fibers was observed to decrease with increasing collector distance. At short distances, the fibers are not completely stabilized and, eventually, the cross-sections of spun fiber become flatter. At long distances, the fibers exhibited a straight, cylindrical morphology, indicating that the fibers are mostly dried when reaching the collector [5]. The properties of the polymer solution, i.e., rheological properties, especially solution viscosity, and electrical conductivity, influence the fiber formation, morphologies, and diameters of the electrospinning process. The boiling point of the solvent is another key factor that influence the diameter. For example, the boiling points of *N*,*N*′-dimethylformamide (DMF) and ethanol were 153 °C and 78 °C, respectively. The lower boiling point solvents undergo the quicker evaporations after the spraying and splitting of an unstable jet [5]. Various polymers, such as poly (vinyl alcohol) (PVA) [6], poly(ethylene oxide) PEO [7,8], and polyacrylonitril (PAN) [9], have been used in the past to examine the effect of fiber formation parameters.

Among these polymers, poly (vinyl pyrrolidone) (PVP) is an important amorphous polymer, with low chemical toxicity, high biocompatiblity, excellent solubility in most organic solvents, good spinnability, and capability to interact with a wide range of hydrophilic materials [4]. For example, Haitao Wang et al. used PVP as an additive to produce polyethersulfone (PES) membrane and the PVP/ PES membrane has higher water adsorption, water flux, and lower water contact angle than the pure PES membrane [10]. Therefore, PVP has been considered as a promising material for potential application in shielding devices, adhesives, detergents, paints, medical devices, and biological engineering materials [5]. In previous studies, uniform electrospun PVP nanofibers without beads were obtained from the polymer solution in DMF in the concentration range of 12–20 wt % [4]. Chitosan was produced by electrospinning of mixed aqueous solutions with PVP [11]. Cellulose acetate (CA) and PVP polymeric fibers were prepared by electrospinning for transdermal patches [12]. Additionally, PVP was also considered as an excellent steric stabilizer or capping agent of various metal nanoparticles in the electrospinning field to protect the product from agglomeration [13]. For instance, gold nanoparticles were prepared by trisodium citrate reduction of HAuCl_4_ in PVP ethanol solution and were dispersed into PVP nanofibers by electrospinning [14]. Silver (Ag) nanoparticles embedded PVP electrospun fibers were successfully prepared, which show good antimicrobial activities [15,16]. However, very little has been reported on the mechanical strength of these fibers as affected by additives.

Due to biodegradability, renewability, low cost, high aspect ratio, high surface area, and high strength, cellulose nanocrystals (CNCs) have been applied as multifunctional agents in various fields, i.e., sound absorption, drug delivery, biosensors, wound dressing [17], drilling fluids [18,19], and reinforced nanocomposites [5,20]. In particular, electrospun PVA [21], poly(lactic acid) (PLA) [22,23], PEO [24,25], poly(ethylene glycol) (PEG) [26], and poly(*ε*-caprolactone) (PCL) [27] composite fibers have been successfully reinforced by CNCs. For example, the PLGA nanofiber membranes reinforced with 7 wt % CNCs had a tensile modulus of 21.28 MPa and an ultimate strain of 89.2% ± 5.3%, which are similar to the values of human skin [28]. The flexural strength and flexural modulus of epoxy were increased by 20% and 17% after adding the CNCs, respectively [20]. With the addition of 5 wt % CNCs, the maximum tensile stress and Young’s modulus of the nanocomposite mats increased by five- and 22-fold than those of neat PLA mats, respectively [29]. The presence of hydroxyl groups in CNCs makes them suitable for the production of composites with polar, hydrophilic polymers, such as PVP. However, PVP/CNC based electrospun fibers, themselves, have no antimicrobial ability.

Silver is toxic to bacteria because of its affinity to proteins and nucleic acids, but non-toxic to human cells [30]. The nanoparticle form of silver is more prone than the bulk material to exhibit novel materials properties. Silver nanoparticles have been extensively studied with regard to their unique physical and chemical properties, which are greatly affected by shape, size, and dispersion stability of particles [31,32]. Nanoparticles could be more efficiently formed to deliver themselves to target organisms [33]. Studiess on the electrospun Ag nanoparticle-embedded polymer nanofibers using the mixture of AgNO_3_ precursor and polymer solutions, i.e., PVA [34], PVP [3,33], poly (lactide-*co*-glycolide) (PLGA) [35], PEO [36,37], and PAN [38,39] exhibited the improved antimicrobial properties in composites. The Tar/PAN/Ag nanofibers showed higher antimicrobial activities (up to 39%) against Gram-positive *Staphylococcus aureus* (*S.* *aureus*) and Gram-negative *Escherichia coli* (*E. coli*) in comparison with the neat PAN nanofibers [39].

The main objectives of this work were to use CNCs as reinforcing agents and Ag nanoparticles as antimicrobial agents in electrospun PVP composite fibers to improve the mechanical properties and antimicrobial properties, respectively. The electrospun precursor suspensions and fibers were characterized in terms of rheology, morphology, thermal properties, and mechanical properties. The influence of CNCs and Ag nanoparticles on morphology and size of the electrospun fibers were characterized. *E. coli* and *S. aureus* were chosen to evaluate the antibacterial activity of electrospun PVP/CNC/Ag composite fibers.

## 2. Experimental

### 2.1. Materials

Poly (vinyl pyrrolidone) (PVP, Mw 40,000 and Mw 360,000), *N*,*N*′-dimethylformamide (DMF), and silver nitrate were purchased from Sigma-Aldrich (St. Louis, MO, USA). All reagents were of analytical grade and were used without further purification.

### 2.2. Preparation of CNCs

The CNCs were isolated from corn stalk using 60 wt % sulfuric acid hydrolysis and mechanical treatments [40]. The prepared CNCs had a needle-like morphology with an average width of 6.4 ± 3.1 nm and length of 120.2 ± 61.3 nm from the transmission electron microscopy (TEM) analysis (see Figure S1 in Supplementary Materials). The aspect ratio was about 18.94.

### 2.3. Preparation of PVP/CNC/Ag Nanocomposites

PVP solution (16.6 wt %) was prepared by dissolving 3 g PVP (Mw = 40,000/360,000 (1:1) g/mol) powder in 15 mL DMF. Then, AgNO_3_ and freeze-dried CNC powder were dispersed in the PVP solution with continuous stirring for 24 h at room temperature to obtain PVP/CNC/AgNO_3_ suspensions with different CNC and Ag ratios. The pure PVP, PVP/CNC-2%, PVP/CNC-4%, PVP/AgNO_3_-0.17%, PVP/AgNO_3_-0.34%, PVP/CNC-2%/AgNO_3_-0.17%, and PVP/CNC-2%/AgNO_3_-0.34% suspensions are designated as Samples 1, 2, 3, 4, 5, 6, and 7 (Table 1), respectively.

Samples from the prepared PVP solution or other suspensions were added to a plastic syringe with a 20 gauge needle (internal diameter = 0.64 mm), which was connected to a high voltage power supply (Gamma High Voltage Research, Ormond Beach, FL, USA). The feeding rate of the polymer was controlled at 1 mL/h by a syringe pump (Chemyx Inc., Stafford, TX, USA). A piece of grounded aluminum foil used as the collector. The distance between the spinneret and collector was 20 cm and the applied voltage was 18 kV. The obtained PVP composites were stored at 22 ± 2 °C and relative humidity (40% ± 2%) before further testing.

## 3. Characterization

### 3.1. Characterization of Electrospinning Suspensions

Electrical conductivity of PVP/CNC, PVP/AgNO_3_, and PVP/CNC/AgNO_3_ suspensions were measured using a Jenway Model 4330 conductivity and PH meter (OAKION, Bath, UK) at room temperature.

The viscosity of prepared suspensions were determined using a rheometer (AR 2000ex, TA Instrument, Inc., New Castle, DE, USA) with a cone and plate geometry (cone angle = 2°; diameter = 40 mm; truncation = 56 μm) at 25 °C. Steady-state viscosity was measured in a shear rate range from 1 to 100 s^−1^.

For non-Newtonian fluids, various mathematical models can be used to fit the relationship between shear stress and shear rate. Among them, the power law and Bingham plastic models are most commonly used to describe this behavior [41,42]. The power law model is widely used for its simplicity:

(1) $$\tau =K\times \gamma^{n}$$

where τ is the shear stress, *K* is the flow consistency coefficient, γ is the shear rate, and n is the flow behavior index. With the power law model, the flow consistency coefficient and flow behavior index can be obtained. However, due to the lack of yield point, the power law model may not be accurate to fit the rheological curves, especially at the low shear rates. To overcome this inconvenience, the Bingham plastic model was considered, as expressed by Equation (2):

(2) $$\tau =\tau_{o}+\mu_{p}\times \gamma$$

where $\mu_{p}$ is the plastic viscosity. With the Bingham plastic model, the yield point and plastic viscosity can be calculated.

### 3.2. Characterization of PVP/CNC/Ag Nanocomposites 

#### 3.2.1. Field Emission-Scanning Electron Microscopy (FE-SEM) Analysis

FE-SEM (FEI Quanta TM 3D FEG dual beam SEM/FIB system, Hillsboro, OR, USA) was used to characterize the surface morphology of the composites. The samples were coated with a thin layer of gold before observation in order to increase the sample conductivity. The diameter and diameter distribution of the fibers in the mats were determined by using Pro Plus 6.3 (Media Cybernetics, Inc., Bethesda, MD, USA) with sampling sizes of at least 100 fibers from FE-SEM micrographs.

#### 3.2.2. Transmission Electron Microscopy (TEM)

To characterize the dispersion of Ag nanoparticles in the PVP electrospun fiber and CNC suspensions, transmission electron microscopy (TEM, JEM 1400, JEOL, Peabody, MA, USA) operating at an accelerating voltage of 120 kV was used. The dimensions of CNCs were measured using the same process. The concentration of CNC suspension was diluted to 0.02% (*w*/*v*) prior to the TEM test.

#### 3.2.3. Fourier Transform Infrared Spectroscopy (FTIR)

An FTIR spectrometer (VERTEX80, Bruker, Billerica, MA, Germany) was used to study the chemical structure of PVP/CNC/Ag composites. The FTIR spectra of materials were evaluated in the range of 4000 to 400 cm^−1^ with a resolution of 4 cm^−1^ at 32 scans.

#### 3.2.4. Thermogravimetric Analysis (TGA)

To study the thermal stability of composite fiber samples, approximately 5 mg of sample were placed in a standard TGA pan and heated in temperature ranging from 30 to 600 °C, with a heating rate of 10 °C/min under a nitrogen flow of 40 cm^3^/min using a thermogravimetric analyzer TAQ50 analyzer (TA Instruments, New Castle, DE, USA).

#### 3.2.5. Mechanical Properties

Tensile strength and elongation at break were measured using the TA AR2000 rheometer (TA Instruments, New Castle, DE, USA) with a solid fixture. Mats were carefully peeled off from the surface of aluminum foil and then placed between two pieces of weighing paper to avoid any direct touch damage on the mat surfaces during sample preparation. The tensile gauge length was 10 mm. The speed of tensile testing was 10 μm/s and three specimens with dimension of 15 mm (length) × 5 mm (width) × 0.2 mm (thickness) were used for each sample group. The stress and strain were calculated through the machine-recorded force and displacement based on the initial cross-section area and gauge length, respectively.

#### 3.2.6. Antimicrobial Performance

Antimicrobial activities of PVP/CNC/Ag composite fibers were tested against both the Gram-negative bacterium *E. coli* and the Gram-positive bacterium *S. aurues* using the Kirby–Bauer antibiotic testing method. First, the bacteria were cultivated in 10 mL sterilized tryptic soy broth and incubated in an incubator at 37 °C. The cell density was monitored by measuring the absorbance at 600 nm of culture medium using a spectrophotometer. When the cell density achieved approximately 10^7^ CFU/mL, the culture medium was taken out from the incubator and carefully spread on the surface of solidified agar plate using a sterile cotton swap. Second, the PVP/CNC/Ag film were cut into disks with a diameter of 12.7 mm and sterilized by UV irradiation for 15 min. The disks were then placed on the inoculated agar plates and incubated at 37 °C for 24 h. Finally, the inhibition zone for bacterial growth was detected visually.

## 4. Results and Discussions

### 4.1. Properties of PVP/CNC/AgNO_3_ Suspension

The solution conductivity plays a key role in the electrospinning process since the viscous polymer solution is stretched due to the repulsion of the charges present on its surface, and more charges can be carried at higher solution conductivity [43]. As listed in Table 1, in comparison with that of the pure PVP solution, the electrical conductivity of PVP/CNC suspensions increased with the addition of CNCs. The conductivity of PVP/CNC-2% and PVP/CNC-4% increased by 9.9 ± 0.17 and 14.9 ± 0.18 µs·cm^−1^ compared with the value of pure PVP, respectively. This was ascribed to the CNC surface having sulfate ester groups and uronic acid [35]. Similarly, PVP/AgNO_3_ suspension also presented higher electrical conductivity than the pure PVP solution because of the excellent electrical conductivity of Ag. The conductivity of PVP/AgNO_3_-0.34% suspension increased from 53.8 ± 0.22 to 59.9 ± 0.23 µs·cm^−1^ in comparison with that of PVP/AgNO_3_-0.17%. The PVP/CNC-4%/AgNO_3_-0.34% suspension had a higher electrical conductivity than other samples.

**Figure 1:**
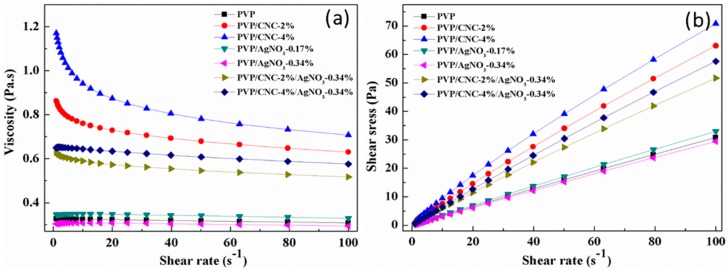
Rheological behavior (**a**) viscosity and (**b**) shear stress vs. shear rate of pure PVP, PVP/AgNO_3_, PVP/CNC, and PVP/CNC/AgNO_3_ suspensions systems.

The viscosity was another critical parameter for determining the morphology of the electrospun fibers. Figure 1a shows the plots of viscosity versus shear rate for the pure PVP, PVP/CNC, PVP/AgNO_3_, and PVP/CNC/AgNO_3_ systems. The rheology behavior of the aqueous PVP solution changed from Newtonian fluid behavior to typical shear thinning behavior after adding the CNCs. The viscosity of suspension at shear rate of 0.1 s^−1^ increased from 0.3 to 1.2 Pa∙s after adding 4% CNCs in the PVP solution, which was due to the growth in the collision possibility of CNCs [19]. Zhang et al. reported that the orientation of macromolecular chains was the major cause of non-Newtonian behavior [44]. With the increase in the shear rate, the number of the oriented polymer segments increased, which decreased the viscosity, greatly promoting the non-Newtonian behavior. However, in comparison with CNCs, Ag^+^ had no effect on the pure PVP solution. Moreover, after adding 0.34 wt % AgNO_3_ to the PVP/CNCs suspension, the viscosity was observed to decrease by 21% and presented a similar Newtonian rheology behavior in comparison with PVP/CNC-2% polymer suspension. The PVP/CNC-4% polymer suspension also had the same tendency after adding AgNO_3_. For PVP/CNC/AgNO_3_ systems, the rheology behavior converted back to the Newtonian fluid and the viscosity was also decreased after adding silver nanoparticles compared with the PVP/CNC systems. This was due to the fact that Ag^+^ destroy some of the hydrogen bonds inside the CNCs, or some bonds between CNCs and PVP, which led to the appearance of competition between CNCs and Ag. All of the results indicated that the combination of CNCs and silver nanoparticles can help adjust the rheology behavior of PVP electrospun systems to meet a desired spinning need.

Figure 1b shows the plots of shear stress versus shear rate for the pure PVP solution, PVP/CNC, PVP/AgNO_3_, and PVP/CNC/AgNO_3_ suspensions. Similar to the viscosity results, the shear stress of the PVP solution also increased with the addition of CNCs. The Bingham plastic and power law models were applied to fit their shear stress-shear rate curves, and the corresponding fit parameters are summarized in Table 2. Both the Bingham plastic and power law models displayed a good fit for the shear stress-shear rate curves. The power law model was evidenced by the higher values of *R*^2^ (>0.99), indicating a good correlation. The difference between power law and Bingham plastic can be explained in terms of yield stress. It can be seen that pure PVP solutions, PVP/CNC-2%, PVP/CNC-4%, PVP/AgNO_3_-0.17%, PVP/AgNO_3_-0.34%, PVP/CNC-2%/AgNO_3_-0.34%, and PVP/CNC-4%/AgNO_3_-0.34% suspensions had yield point values of 0.127, 1.011, 1.813, 0.149, 0.105, 0.556, and 0.520 Pa∙s from the Bingham plastic model, respectively. The PVP systems with CNCs had higher yield point values than pure PVP and PVP/AgNO_3_ suspensions systems. The yield point, the stress required to move the electrospun suspensions, plays an important role in the electrospinning process, which can be used to predict the feeding rate and the morphology of electrospun fibers. The flow behavior index describes the type of suspensions: Newtonian (*n* = 1), non-Newtonian with a shear thinning behavior (*n* < 1), or non-Newtonian with shear thickening behavior (*n* > 1) [45]. From this aspect, the flow behavior index of PVP, PVP/AgNO_3_-0.17%, and PVP/AgNO_3_-0.34% was near 1, indicating the Newtonian fluid behavior. The addition of CNCs in the suspension led to increased shear thinning behavior (reduced *n* value).

### 4.2. Morphologies of Fibrous Mats

**Figure 2:**
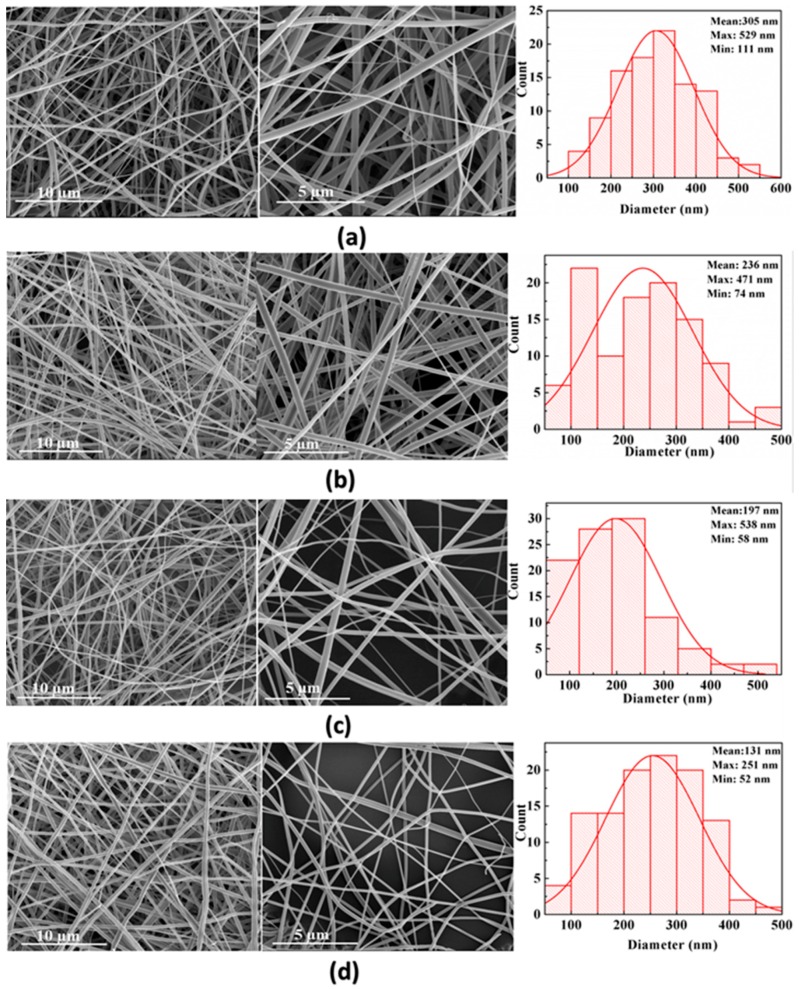
The representative FE-SEM images and corresponding fiber diameter distribution of electrospun fibers: (**a**) PVP; (**b**) PVP/CNC-2%; (**c**) PVP/CNC-4%; and (**d**) PVP/CNC-4%/AgNO_3_-0.34%. Each subfigure shows spun fibers at 10 μm and 5 μm resolution levels, and diameter distribution (bar chart).

Figure 2 shows the morphology of electrospun neat PVP (Figure 2a), PVP/CNC-2% (Figure 2b), PVP/CNC-4% (Figure 2c), and PVP/CNC-4%/AgNO_3_-0.34% (Figure 2d) composite fibers. The average fiber diameter (AFD) of PVP, PVP/CNC-2%, PVP/CNC-4%, PVP/AgNO_3_-0.17%, PVP/AgNO_3_-0.34%, PVP/CNC-2%/AgNO_3_-0.34%, and PVP/CNC-4%/AgNO_3_-0.34% were, respectively, 305 ± 31, 236 ± 40, 197 ± 41, 214 ± 35, 193 ± 43, 151 ± 45, and 131 ± 46 nm (Table 1). With the increase in the silver nanoparticle content, the AFD of PVP/AgNO_3_-0.17%, PVP/AgNO_3_-0.34%, PVP/CNC-2%/AgNO_3_-0.34%, and PVP/CNC-4%/AgNO_3_-0.34% composite fibers decreased to 214 ± 35, 193 ± 43, 151 ± 45, and 131 ± 46 nm (Table 1), respectively. In comparison with pure PVP, the conductivity of PVP/AgNO_3_-0.34% increased by 32%, while the AFD decreased by 37% (Table 1). The increased electrical conductivity led to increased surface charge of the polymer jet, and thus, stronger elongation forces were imposed to the jet, resulting in defect-free, more uniform fibers with a thinner diameter distribution [29,37]. Based on the conductivity and viscosity of solution above, it was concluded that PVP/CNC-4%/AgNO_3_-0.34% had the smaller diameter owing to the higher conductivity and controllable rheological properties. This phenomenon was similar to that of the electrospun Ag/CS/PEO and Ag/CNC/PLA fibers study by Jing An and Cacciotti et al. [36,46].

The FE-SEM-EDS data were recorded in order to provide further confirmation on the formation of silver nanoparticles on cellulose fibers. SEM-EDS spectra of silver nanoparticles impregnated composite are presented as shown in Figure S2 (Figure S2 in Supplementary Materials). The obtained EDS spectrum of silver nanoparticles impregnated PVP/CNC-4%/AgNO_3_-0.34% confirms the existence of silver nanoparticles in the PVP/CNC-4%/AgNO_3_-0.34%, amounting at 0.37 wt %. The electrospinning process favors the uniform dispersion of Ag^+^ species in PVP chains through the interaction with the carbonyl groups in the PVP molecules [13].

**Figure 3:**
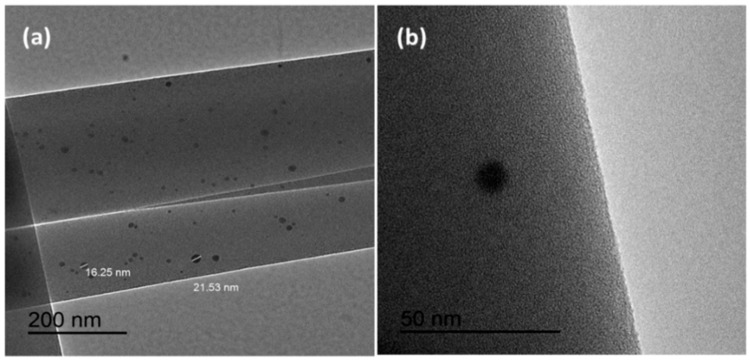
TEM micrographs of electrospun fibers: (**a**,**b**) PVP/CNC-4%/AgNO_3_-0.34%.

Figure 3 shows the TEM micrographs of electrospun PVP/CNC/AgNO_3_-0.34% fibers. Most Ag nanoparticles on the electrospun fibers had diameters between 7.84 and 21.53 nm (Figure 3a). It was clearly observed that individual fibers contained Ag nanoparticles on the surface of the fibers (Figure 3b). The Ag nanoparticles dispersed well in the electrospun composited fibers. The PVP not only promoted the nucleation of Ag nanoparticles, but also prohibited their aggregation [47]. Compared with other PVP-based composites, such as PVP/polyaniline (PANI), the aggregation between PANI was also significantly reduced by using PVP as the dispersing medium and, hence, the storage stability of the dispersions was improved as compared with a direct dispersion of PANI in distilled water [48].

### 4.3. FTIR Analysis

**Figure 4:**
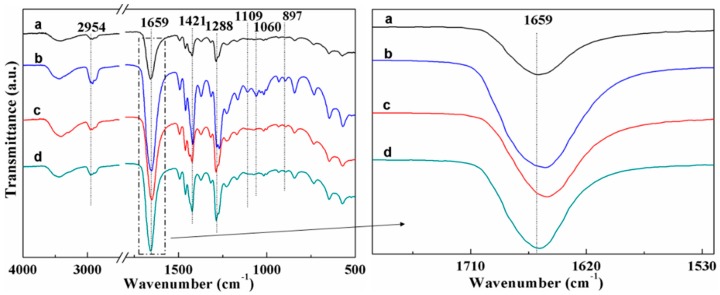
FTIR spectra of: (**a**) PVP; (**b**) PVP/CNC-4%; (**c**) PVP/AgNO_3_-0.34%; and (**d**) PVP/CNC-4%/AgNO_3_-0.34%.

Figure 4 shows the FTIR spectra of PVP, PVP/CNC-4%, PVP/AgNO_3_-0.34%, and PVP/CNC-4%/AgNO_3_-0.34%. The peaks located at 2954, 1654, 1421, and 1288 cm^−1^ for PVP (Figure 4a) were assigned to the stretching vibrations of C–H, C=O, C=C, and C–N, respectively [15]. The characteristic bands such as C–O stretching at 1060 cm^−1^, C–H rock at 897 cm^−1^, and C–OH stretching at 1109 cm^−1^ are the spectrum of cellulose after acid hydrolysis [49,50,51]. These peaks were all observed in the blends of PVP/CNC-4%, indicating that the PVP/CNC-4% fiber composites contained both PVP and CNCs. With addition of 4 wt % CNCs, the absorption band of PVP at 1659 cm^−1^ shifted to 1651 cm^−1^ (Figure 4), which indicated the presence of some molecular interaction between PVP and CNCs. After the AgNO_3_ was added, the absorption band of PVP at 1659 cm^−1^ shifted to 1648 cm^−1^ (Figure 4), suggesting the presence of some interaction between Ag^+^ and the C=O groups [34,52]. A similar result that involves Ag and PVP has also been reported by Chen et al. [53]. However, when 4 wt % CNCs were added to the PVP/AgNO_3_-0.34% composites, the band of PVP/AgNO_3_-0.34% at 1648 cm^−1^ shifted back to 1659 cm^−1^ again. This was ascribed to the existence of Ag disturbing the hydrogen bonds in the network structure of CNCs. Then, the characteristic peaks of CNCs, such as C–O stretching at 1060 cm^−1^, C-H rock at 897 cm^−1^, and C–OH stretching at 1109 cm^−1^, become weak. The hydrogen bonds of CNCs crosslinked the Ag, which could help better disperse CNCs in the polymer suspensions. This was why the non-Newtonian behavior of PVP/CNC converted to Newtonian behavior after adding the silver compound.

### 4.4. Thermal Properties

**Figure 5:**
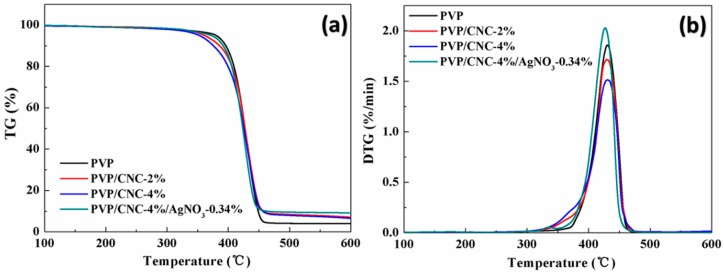
TG (**a**) and DTG (**b**) curves of the electrospun neat PVP, PVP/CNC-2%, PVP/CNC-4%, and PVP/CNC-4%/AgNO_3_-0.34% fibers.

TGA and derived differential TG (DTG) curves of neat PVP, PVP/CNC-2%, PVP/CNC-4%, and PVP/CNC-4%/AgNO_3_-0.34% mats are shown in Figure 5a and Figure 5b, respectively. Thermal parameters, including onset thermal degradation temperature (*T*_10*%*_) and the maximum thermal degradation temperature (*T_max_*), are summarized in Table 3. Herein, the onset thermal degradation temperature is regarded as the temperature corresponding to 10% weight loss (*T*_10*%*_). *T*_10*%*_ of neat PVP was 396.0 °C, while the addition of CNCs decreased the *T*_10*%*_ to 385.8 (2 wt % of CNCs) and 375.8 °C (4 wt % of CNCs), respectively. The reason was that, in comparison with PVP, neat CNCs had a lower *T*_10*%*_ (Table 3), which indicated the relatively lower thermal stability. However, with addition of silver nanoparticles, the *T*_10*%*_ values of PVP/CNC-4%/AgNO_3_-0.34% nanocomposite mats increased to 391.6 °C, indicating that its heat resistance increased. The addition of inorganic materials always increases thermal stability of polymers [54]. The *T_max_* of PVP/CNC and PVP/CNC-4%/AgNO_3_-0.34% mats almost had no changes, compared to that of pure PVP. Figure 5a also displays the char yield of electrospun PVP/CNC-4%/AgNO_3_-0.34% nanocomposite fibers increased with the addition of silver nanoparticles. Increased char formation can limit the production of combustible gases, decrease the exothermicity of the pyrolysis reaction, and inhibit the thermal conductivity of the burning materials [29].

### 4.5. Mechanical Properties

**Figure 6:**
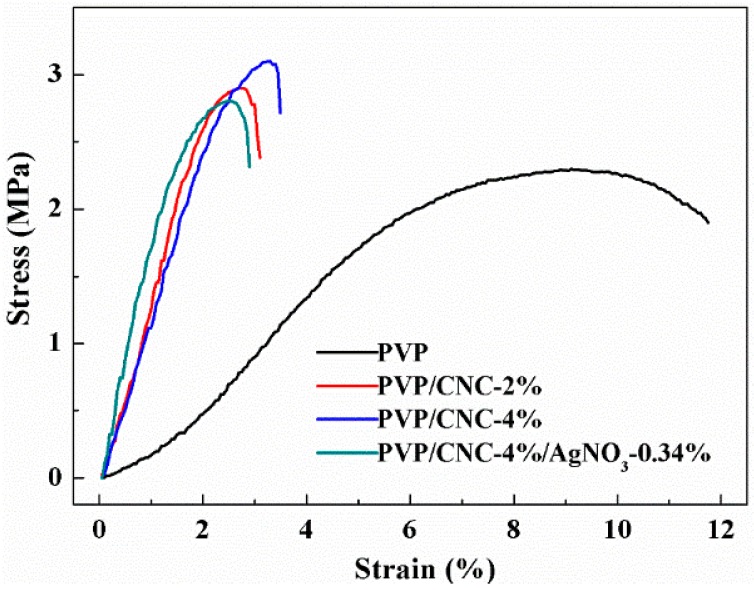
Typical stress-strain curves of electrospun pure PVP, PVP/CNC-2%, PVP/CNC-4%, and PVP/CNC-4%/AgNO_3_-0.34% composite fiber mats.

Figure 6 shows stress-strain curves of electrospun pure PVP, PVP/CNC-2%, PVP/CNC-4%, and PVP/CNC-4%/AgNO_3_-0.34% composite fiber mats. The values of the elongation at break and ultimate tensile strength are summarized in Table 4. Upon the addition of 4 wt % CNCs, the ultimate tensile strength of pure PVP increased by approximately 0.8 MPa (from 2.30 ± 0.2 to 3.10 ± 0.1 MPa), indicating a reinforcing effect of the filler. However, with addition of CNCs, the elongation at break decreased sharply, which suggests that the composite became brittle in comparison with pure PVP. A similar result was reported by Zhang et al. regarding the PLA/CNC composite fibers [55]. For electrospun composite fibers, mechanical properties could be affected by several factors, i.e., individual fiber structure, molecular alignment of the amorphous chains, fiber alignment, and inter-fiber bonding [56]. With the addtion of 0.34 wt % AgNO_3_, the elongation at break and ultimate tensile strength decreased slightly. This was due to the addtion of Ag nanoparticles, leading to the presence of stress concentration in the nanofiber membranes [15].

### 4.6. Antimicrobial Performance

**Figure 7:**
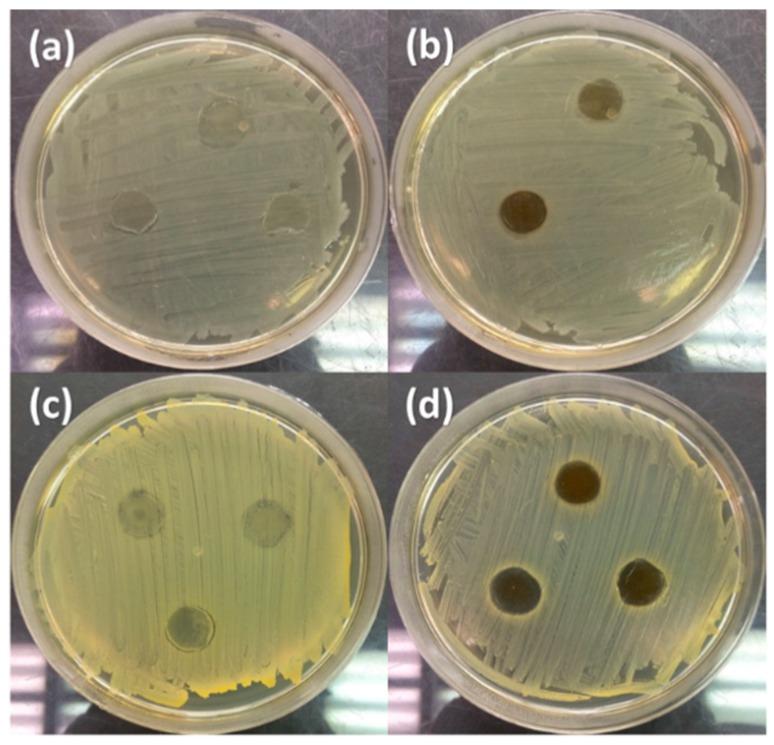
Photographs of the inhibition zones of PVP/CNC against *E. coli* (**a**) and *S. aureus* (**c**); and PVP/CNC-4%/AgNO_3_-0.34% against *E. coli* (**b**), and *S. aureus* (**d**).

The antimicrobial properties of PVP/CNC-4%/AgNO_3_-0.34% composites were tested against *E. coli* and *S. aureus* bacteria by disk diffusion testing. The inhibition zones are presented in Figure 7. After 24 h of incubation, there was bacterial growth directly under PVP/CNC, and also up to the edge of the fabric for both *E. coli* and *S. aureus*. However, PVP/CNC-4%/AgNO_3_-0.34% composites fibers acted as an excellent antimicrobial agent against both *E. coli* and *S. aureus*. This could be ascribed to the antimicrobial feature of Ag^+^. Ag particles released from PVP/CNC-4%/AgNO_3_-0.34% composite fibers [57] and the Ag nanoparticles attached to the cell walls and disturbed cell wall permeability and cellular respiration [15]. As the results shown above, the electrospun PVP/CNC-4%/AgNO_3_-0.34% composite fibers had a good potential for application as antimicrobial materials.

## 5. Conclusions

With the addition of CNCs and Ag nanoparticles, the PVP/CNC/Ag electrospun suspensions exhibited higher conductivity and controllable rheological properties (viscosity, shear stress, and yield point) using DMF as the solvent. Only a small amount of CNCs and Ag can help tune rheological properties and electrospinning ability. Both rheological properties and FTIR spectra indicated that the existence of Ag disturbed the hydrogen bonds in the network structure of CNCs. FE-SEM results show that the diameter of composite fibers was uniform. The average diameter of the electrospun fibers decreased with the increased loading of CNCs and Ag nanoparticles. Most Ag nanoparticles in the electrospun fibers had diameters between 7.84 and 21.53 nm. The CNCs helped increase the values of tensile strength slightly, while the value of elongation at break decreased. Thermal stability of the composite fibers decreased slightly with the addition of CNCs, but then increased with the presence of Ag nanoparticles. The PVP/CNC/Ag composites fibers showed improved antibacterial activity against both *E. coli* and *S. aureus* than the PVP/CNC composite fibers. The aggregation between Ag nanoparticles was significantly reduced by using PVP as the dispersing medium through electrospinning technology. This indicated that the controllable size of Ag nanoparticles was of potential use in the antibacterial materials at room temperature.

## Figures and Tables

**Table 1:** Electrical conductivity of electrospinning pure PVP, PVP/AgNO_3_, PVP/CNC, and PVP/CNC/AgNO_3_ systems.

Samples	Composition	Electrical Conductivity (µs·cm^−1^)	Viscosity (Pa·s)	Average Fiber Diameters (nm)
1	PVP	45.3 ± 0.17	0.3228	305 ± 31
2	PVP/CNC-2%	55.2 ± 0.19	0.7185	236 ± 40
3	PVP/CNC-4%	60.2 ± 0.20	0.8512	197 ± 41
4	PVP/AgNO_3_-0.17%	53.8 ± 0.22	0.3467	214 ± 35
5	PVP/AgNO_3_-0.34%	59.9 ± 0.23	0.3075	193 ± 43
6	PVP/CNC-2%/AgNO_3_-0.34%	84.8 ± 0.23	0.5675	151 ± 45
7	PVP/CNC-4%/AgNO_3_-0.34%	157.7 ± 0.24	0.6228	131 ± 46

**Table 2:** Calculated parameters for pure PVP solution, PVP/AgNO_3_, PVP/CNC, and PVP/CNC/AgNO_3_ suspensions systems using Bingham plastic (BP), and power law (PL) models.

Samples	Models					
	BP			PL		
	τ_o_	μ	*R*^2^	*K*	*n*	*R*^2^
PVP	0.127	0.311	0.9997	0.353	0.97	0.9999
PVP/CNC-2%	1.011	0.639	0.9980	0.951	0.91	0.9999
PVP/CNC-4%	1.813	0.717	0.9962	1.280	0.87	0.9999
PVP/AgNO_3_-0.17%	0.149	0.333	0.9996	0.384	0.97	0.9999
PVP/AgNO_3_-0.34%	0.105	0.297	0.9997	0.334	0.97	0.9999
PVP/CNC-2%/AgNO_3_-0.34%	0.556	0.523	0.9989	0.694	0.94	0.9999
PVP/CNC-4%/AgNO_3_-0.34%	0.520	0.584	0.9990	0.753	0.94	0.9999

**Table 3:** Summary of TGA and DTG curves of electrospun PVP, PVP/CNC-2%, PVP/CNC-4%, and PVP/CNC-4%/AgNO_3_-0.34% composite fiber mats.

Composition	*T*_10*%*_ (°C)	*T_max_* (°C)	Residue (%)
PVP	396.0	430.5	4.0
PVP/CNC-2%	385.8	429.3	7.1
PVP/CNC-4%	375.8	431.0	6.6
PVP/CNC-4%/AgNO_3_-0.34%	391.6	426.8	9.2
CNC	292.5	348.3	–

**Table 4:** Tensile properties of electrospun pure PVP, PVP/CNC-2%, PVP/CNC-4%, and PVP/CNC-4%/AgNO_3_-0.34% composite fiber mats.

Sample	Elongation at Break (%)	Ultimate Tensile Strength (MPa)
PVP	9.10 ± 0.2	2.30 ± 0.2
PVP/CNC-2%	2.70 ± 0.2	2.90 ± 0.2
PVP/CNC-4%	3.25 ± 0.2	3.10 ± 0.1
PVP/CNC-4%/AgNO_3_-0.34%	2.50 ± 0.3	2.81 ± 0.3

## Supplementary Materials

The following are available online at www.mdpi.com/1996-1944/9/7/523/s1.
