# Supplementary material for: Preparation and Properties of Electrospun Poly (Vinyl Pyrrolidone)/Cellulose Nanocrystal/Silver Nanoparticle Composite Fibers
Source: Materials (Basel). 2016 Jun 28;9(7):523. doi: 10.3390/ma9070523 (PMC5456859; doi:10.3390/ma9070523)
Supplement: Supplementary file 1 [file materials-09-00523-s001.pdf]

Supplementary Materials: Preparation and Properties of Electrospun Poly (Vinyl Pyrrolidone)/Cellulose Nanocrystal/Silver Nanoparticle Composite Fibers

Siwei Huang, Ling Zhou, Mei-Chun Li, Qinglin Wu, Yoichi Kojima and Dingguo Zhou

The extraction of cellulose nanocrystals (CNCs) from corn stalk included several steps. First, 10 g of raw materials were treated by Soxhlet extraction for 7 h using an ethanol/benzene (1:2 *v*/*v*) mixed solvent. Second, the extracted fibers were soaked in acidic sodium chlorite ($NaClO_2$) solution for 1 h at 75 °C to remove lignin. The delignification step was repeated two times to obtain white products. Third, the delignified product was treated with 8 wt % NaOH solution at liquid-solid mass ratio of 20/1 for 2 h at 80 °C. The alkali-treated pulp was washed several times with distilled water until the pH reached neutral. Finally, the pulp was hydrolyzed with 60 wt % sulfuric acid at acid-pulp ratio of 20/1 for 1 h at 60 °C with stirring and diluted using 10-fold deionized water. The diluted suspension was centrifuged at 4000 rpm for 5 min to obtain precipitates. The centrifuged treatment was repeated two times to reduce the acid content. The resultant precipitate was washed, recentrifuged, and dialyzed against deionized water for two days until a constant neutral pH was achieved. The obtained CNCs had a need-like morphology with an average width of 6.4 ± 3.1 nm and length of 120.2 ± 61.3 nm. The aspect ratio was about 18.94.

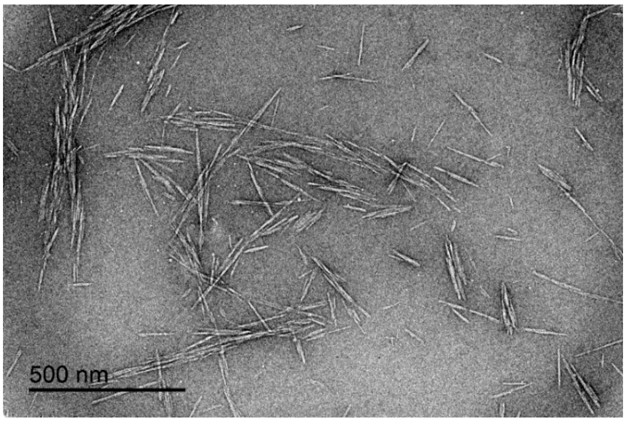

Figure S1. TEM micrographs of CNCs suspensions.

The FE-SEM-EDS data was recorded in order to provide further confirmation on the formation of silver nanoparticles on cellulose fibers. SEM-EDS spectra of silver nanoparticle-impregnated composite are presented in Figure S2. The obtained EDS spectrum of silver nanoparticles impregnated PVP/CNC-4%/$AgNO_3$-0.34% confirms the existence of silver nanoparticles in the PVP/CNC-4%/$AgNO_3$-0.34%, amounting at 0.37 wt %. The electrospinning process favors the uniform dispersion of Ag^+ species in PVP chains through the interaction with the carbonyl groups in the PVP molecules [1].

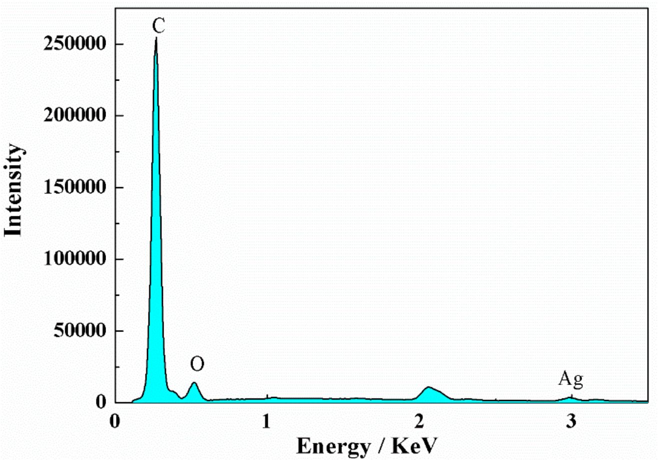

Figure S2. SEM-EDS spectra of electrospun PVP/CNC-4%/$AgNO_3$-0.34% fibers.

Reference

1. Bai, J.; Li, Y.; Li, M.; Wang, S.; Zhang, C.; Yang, Q. Electrospinning method for the preparation of silver chloride nanoparticles in PVP nanofiber. *Appl. Surf. Sci.* **2008**, *254*, 4520–4523.
